# Facile preparation of multifunctional superparamagnetic PHBV microspheres containing SPIONs for biomedical applications

**DOI:** 10.1038/srep23140

**Published:** 2016-03-23

**Authors:** Wei Li, Jan Zaloga, Yaping Ding, Yufang Liu, Christina Janko, Monika Pischetsrieder, Christoph Alexiou, Aldo R. Boccaccini

**Affiliations:** 1 Institute of Biomaterials, Department of Materials Science and Engineering, University of Erlangen-Nuremberg, Cauerstrasse 6, 91058 Erlangen, Germany; 2 Department of Otorhinolaryngology, Head and Neck Surgery, Section for Experimental Oncology and Nanomedicine (SEON), Else Kröner-Fresenius-Stiftung Professorship, University Hospital Erlangen, Glückstrasse 10a, 91054 Erlangen, Germany; 3 Institute of Polymer Materials, Department of Materials Science and Engineering, University of Erlangen-Nuremberg, Martensstrasse 7, 91058 Erlangen, Germany; 4 Henriette Schmidt-Burkhardt Chair of Food Chemistry, Department of Chemistry and Pharmacy, University of Erlangen-Nuremberg, Schuhstrasse19, 91052 Erlangen, Germany

## Abstract

The promising potential of magnetic polymer microspheres in various biomedical applications has been frequently reported. However, the surface hydrophilicity of superparamagnetic iron oxide nanoparticles (SPIONs) usually leads to poor or even failed encapsulation of SPIONs in hydrophobic polymer microspheres using the emulsion method. In this study, the stability of SPIONs in poly(3-hydroxybutyrate-co-3-hydroxyvalerate) (PHBV) solution was significantly increased after surface modification with lauric acid. As a result, magnetic PHBV microspheres with high encapsulation efficiencies (71.0–87.4%) were prepared using emulsion-solvent extraction/evaporation method. Magnetic resonance imaging (MRI) showed significant contrast for the magnetic PHBV microspheres. The toxicity of these magnetic PHBV microspheres towards human T-lymphoma suspension cells and adherent colon carcinoma HT-29 cells was investigated using flow cytometry, and they were shown to be non-toxic in a broad concentration range. A model drug, tetracycline hydrochloride, was used to demonstrate the drug delivery capability and to investigate the drug release behavior of the magnetic PHBV microspheres. The drug was successfully loaded into the microspheres using lauric acid-coated SPIONs as drug carrier, and was released from the microspheres in a diffusion controlled manner. The developed magnetic PHBV microspheres are promising candidates for biomedical applications such as targeted drug delivery and MRI.

Magnetic polymer microspheres have attracted increasing attention and are being widely used in biomedical fields such as drug delivery^1^,^2^,^3^,^4^, magnetic resonance imaging (MRI)^2^,^3^,^4^,^5^,^6^, bio-separation^7^,^8^, enzyme immobilization^9^, hyperthermia therapy^10^ as well as water treatment^11^. Particularly, magnetic polymer microspheres have the advantage of being readily multifunctional, such as enabling the targeted drug delivery procedure being monitored by MRI^2^,^3^,^12^. Magnetically targeted drug delivery has emerged as a promising strategy to deliver drugs to the site of interest using an external magnetic field^1^,^2^,^3^,^13^. Local drug concentrations can be enhanced over 50-fold compared to standard intravenous application^14^. Therefore, in this superior drug delivery system, the amount of circulating drug can be reduced by the control of magnetically targeted drug, reducing toxicity and side effects after systemic administration. Moreover, when polymer microspheres are used for magnetic targeted drug delivery, the polymer matrix has the potential to protect the drug from degradation.

The success of these technologies using magnetic microspheres greatly depends on their preparation from biocompatible and biodegradable polymers of either synthetic^2^,^8^,^15^,^16^,^17^ or natural origin^3^,^12^,^18^. To date, magnetic polymer microspheres have been prepared by various methods, such as emulsion-solvent extraction/evaporation^2^,^15^,^19^, spray drying^1^, electrospraying^17^, polymerization^8^ and microfluidics^3^, which feature partly competing and partly complementary characteristics. Among these methods, the emulsion-solvent extraction/evaporation method possesses significant competitive advantages including good reproducibility and high degree of control over particle characteristics such as particle size, which make it one of the most popular methods to produce microspheres^20^,^21^.

Iron oxide nanoparticles, the only Food and Drug Administration (FDA)- and European Medicines Agency (EMA)-approved metal oxide nanoparticles, have attracted tremendous attention in targeted drug delivery, MRI and hyperthermia therapy^13^. The surface hydrophilicity of iron oxide nanoparticles enables them to be efficiently encapsulated by hydrophilic polymers, which in most cases are natural derived polymers^3^,^11^,^18^. However, the hydrophilic iron oxide nanoparticles strongly tend to partition into the external aqueous phase during emulsification, which leads to great loss of iron oxide nanoparticles or even failure in preparing hydrophobic polymer-based magnetic microspheres^2^,^15^. Most of the hydrophobic polymers are synthetic derived. Compared to natural polymers, synthetic polymers often offer better control of physicochemical properties^22^. Obviously, a good stability of iron oxide nanoparticles in hydrophobic polymer solutions is a prerequisite to the successful fabrication and expanding application of magnetic polymer microspheres. Surface modification with fatty acids has been shown to improve the stability of iron oxide nanoparticles in dichloromethane (DCM)^23^, which is a typical organic solvent used to dissolve hydrophobic polymers.

In the present study, superparamagnetic iron oxide nanoparticles (SPIONs) were surface modified with lauric acid, which belongs to the family of fatty acid. It was hypothesized that lauric acid could enable the SPIONs to be stable in hydrophobic polymer solution, and therefore facilitate the successful preparation of magnetic polymer microspheres with high encapsulation efficiency/loading efficiency using emulsion-solvent extraction/evaporation method. Lauric acid was used in the present study also because the derived lauric acid-modified SPIONs have great potential for magnetic drug targeting, as shown in a previous study^14^,^24^. Poly(3-hydroxybutyrate-co-3-hydroxyvalerate) (PHBV), which belongs to the class of polyhydroxyalkanoates (PHAs), is a promising biotechnology derived hydrophobic polymer used for biomedical applications due to its biocompatibility, nontoxic and tailorable biodegradability^25^,^26^. In addition, unlike poly(lactic acid) (PLA) and poly(lactic-co-glycolic acid) (PLGA), PHBV does not produce acidic degradation products which may be harmful for human tissues, and it degrades into its monomers *in vivo* which are normal in the blood^27^.

In the first part of this investigation, SPIONs were surface modified with lauric acid, and then encapsulated into PHBV microspheres using solid-in-oil-in-water (S/O/W) emulsion-solvent extraction/evaporation method. The physicochemical properties and *in vitro* biocompatibility of the prepared magnetic PHBV microspheres were investigated. It should be pointed out that although various types of magnetic polymer microspheres have been developed^1^,^2^,^3^,^8^,^11^,^15^,^16^,^17^,^19^, only few *in vitro* or *in vivo* studies have been reported on these systems^1^,^2^,^3^,^4^. Thus, there is a strong demand to understand the biocompatibility of magnetic polymer microspheres, and the influence that polymer encapsulation may have on the toxicity of SPIONs. PHAs based-magnetic particles (from nm to mm size range) have only been reported recently^28^,^29^, and their biocompatibility has not been investigated yet to the best of our knowledge. Furthermore, in the present study, tetracycline hydrochloride (TCH), a widely used antibiotic, was chosen as a model of water-soluble drugs to demonstrate the drug delivery capability and to investigate the drug release behavior of the magnetic PHBV microspheres *in vitro*.

## Results and Discussion

### Surface modification on SPIONs^0^

**Figure 1:**
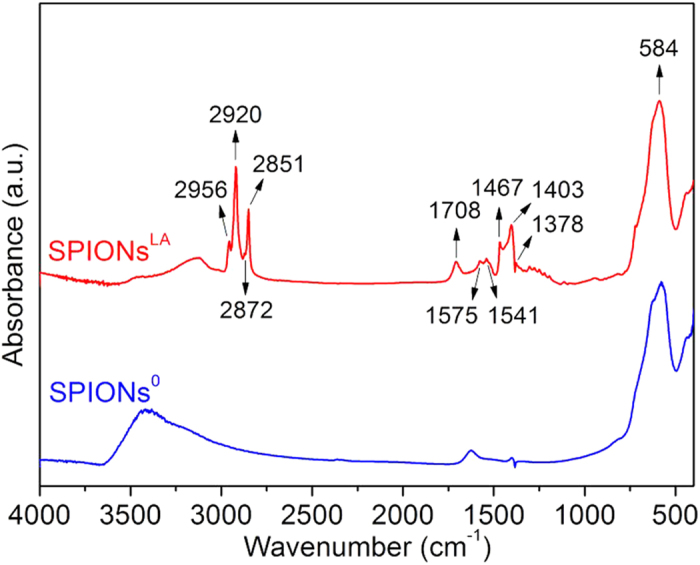
FTIR spectra of SPIONs before (SPIONs^0^) and after (SPIONs^LA^) surface modification with lauric acid. (The different relevant peaks are explained in the text).

The Fourier transform infrared spectroscopy (FTIR) spectra of the two investigated iron oxide nanoparticles SPIONs^0^ (without any coating) and SPIONs^LA^ (with lauric acid coating) are shown in Fig. 1. The characteristic band of Fe–O stretching vibration was observed at 584 cm^−1^ for both SPIONs^0^ and SPIONs^LA^^16^,^24^. All the labelled bands of SPIONs^LA^ except 584 cm^−1^, 1541 cm^−1^ and 1575 cm^−1^ belong to lauric acid^24^,^30^. In addition, compared to the other bands of (free) lauric acid, the band at 1708 cm^−1^ which belongs to carboxylic acid groups was relatively weak in SPIONs^LA^, and new bands at 1541 cm^−1^ and 1575 cm^−1^, which were attributed to carboxylate groups, appeared in SPIONs^LA^^31^,^32^. The appearance of carboxylate groups as well as weakened carboxylic groups indicates that chemical bonds formed between lauric acid and iron oxide nanoparticles^30^,^32^. Therefore, it is confirmed that lauric acid has been successfully coated on SPIONs^0^.

**Figure 2:**
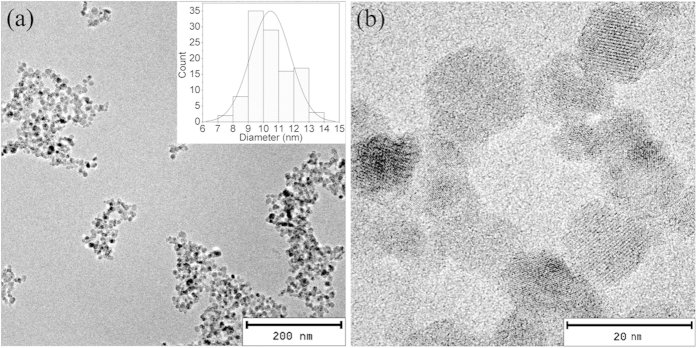
TEM images of SPIONs^LA^ at different magnifications (a,b). The inset in (**a**) shows the particle size distribution of iron oxide cores.

SPIONs^LA^ are quite homogeneous as shown by the transmission electron microscopy (TEM) image (Fig. 2(a)). The diameter of the iron oxide cores was 11 ± 1 nm, which is much smaller than the superparamagnetic critical size (20 nm) of Fe_3_O_4_ nanoparticles^33^. At a higher magnification (Fig. 2(b)), lattice planes of individual particles are visible. The small and homogeneous particle size of SPIONs^LA^ is expected to enhance their stability in hydrophobic polymer solution.

**Figure 3:**
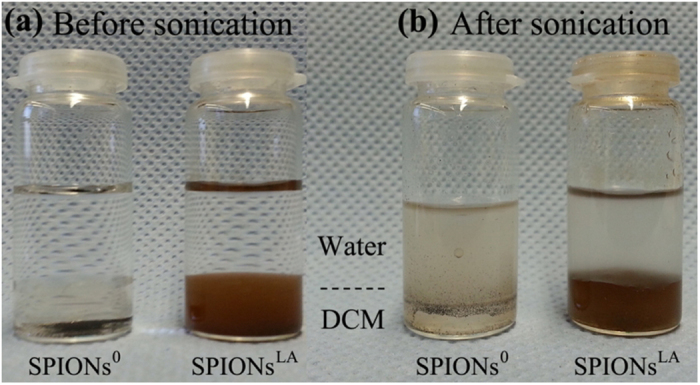
Photos of SPIONs^0^ and SPIONs^LA^ in water/DCM: (a) just after adding DCM and water, (b) 2 min after sonication.

It is of great importance to increase the stability of SPIONs (Solid (S) phase) in PHBV–DCM solution (Oil (O) phase), because a stable S/O solution can reduce the loss of S phase during emulsification, which is a prerequisite to obtain a high encapsulation efficiency. In addition, a stable S/O droplet facilitates the production of more spherical shaped particles after solidification. As shown in Fig. 3(a), SPIONs^0^ sink to the bottom of the DCM, while SPIONs^LA^ already partly disperse in the DCM even without sonication. After sonication (Fig. 3(b)), SPIONs^LA^ are still stably present in the DCM, however most of the SPIONs^0^ were located in the water/DCM interface or inside the water. This result confirms that hydrophilic SPIONs^0^ turn hydrophobic after surface modification with lauric acid, which therefore enables the SPIONs^LA^ to be stable in DCM, with the primary monolayer of lauric acid acting as the stabilizing layer^34^.

### Composition of magnetic PHBV microspheres

**Figure 4:**
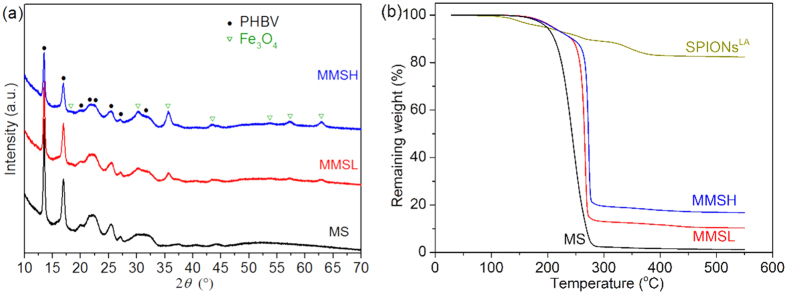
(**a**) XRD patterns and (**b**) TGA of MS (pure PHBV microspheres) and MMSL, MMSH (magnetic PHBV microspheres). TGA of SPIONs^LA^ was performed for comparison.

The main composition of the pure PHBV microspheres (MS) and magnetic PHBV microspheres loaded with different content of SPIONs^LA^ (MMSL (low content) and MMSH (high content)) was investigated by X-ray diffraction (XRD) analysis (Fig. 4(a)). All the samples showed characteristic peaks of PHBV at 13.5°, 16.9°, 19.9°, 21.6°, 22.8°, 25.6°, 27.2° and 30.8°^35^. Both MMSL and MMSH showed the characteristic peaks of Fe_3_O_4_ at 18.2°, 30.2°, 35.6°, 43.3°, 53.7°, 57.2° and 63.1°^36^, and the intensities of these peaks increased when the amount of loaded SPIONs^LA^ was increased. The XRD patterns confirm that Fe_3_O_4_ is successfully encapsulated in the PHBV microspheres.

The thermogravimetric behaviors of SPIONs^LA^ and microspheres are shown in Fig. 4(b). The weight loss of SPIONs^LA^ was mainly due to the decomposition of lauric acid. Compared to SPIONs^LA^, all the microspheres presented much higher weight loss due to the decomposition of PHBV. The thermogravimetric analysis (TGA) curves of the microspheres containing more SPIONs^LA^ shifted towards higher temperature. This may be due to the increased thermal stability of PHBV-based nanocomposites in the presence of more nanoparticles^35^.

The loading efficiency of SPIONs^LA^ in MMSL and MMSH was calculated to be 12.5 wt% and 20.3 wt%, and the encapsulation efficiency was 87.4% and 71.0%, respectively. The density of Fe_3_O_4_ (~5 g/cm^3^) is much higher than that of PHBV (~1.26 g/cm^3^) and DCM (~1.33 g/cm^3^). Therefore, more SPIONs^LA^ loaded in the PHBV–DCM (i.e., higher loading efficiency) may increase the instability of the emulsion which eventually leads to more SPIONs^LA^ been lost, i.e., lower encapsulation efficiency. SPIONs have been reported to be encapsulated in various polymer microspheres, while their encapsulation efficiency, as an important evaluation index of magnetic microsphere, has been reported in only few studies^1^,^2^,^4^,^15^,^17^,^19^,^37^. The encapsulation efficiency in the present study is similar to that (estimated to be ~65−84%) of PLGA/PLA-PEG magnetic microspheres produced by O/W single emulsion method using hydrophobic magnetite gel as the magnetic component^19^, and is significantly higher than that (1.8−4.5%) of hydrophilic ferrofluid loaded PLGA microspheres produced by conventional W/O/W double emulsion method^2^,^4^. In addition, the encapsulation efficiency achieved in this study is higher than that (60%) of hydrophilic F_3_O_4_ nanoparticles loaded PLA microspheres produced by a delicate W/O/W double emulsion method^15^.

### Surface morphology and internal structure

**Figure 5:**
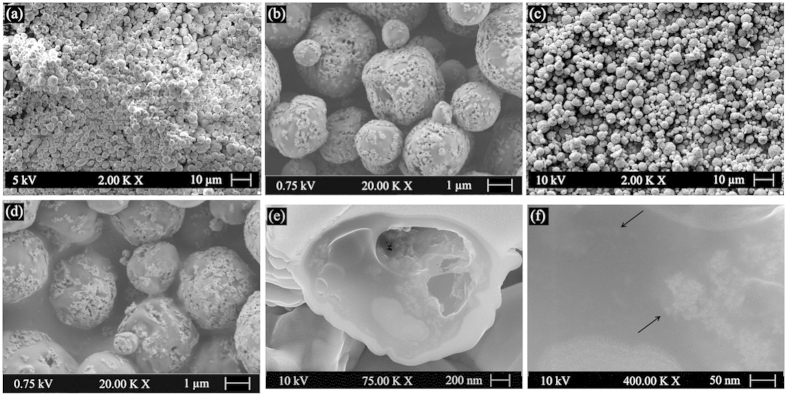
SEM images of magnetic PHBV microspheres at different magnifications: surface morphology of (a,b) MMSL and (c,d) MMSH, and internal microstructure of (e,f) MMSH.

The shape and surface morphology of the microspheres, as observed by scanning electron microscopy (SEM), are shown in Fig. 5. Both MMSL and MMSH appeared spherical in shape, and the microspheres had quite homogeneous particle size. Many SPIONs^LA^ were seen to be embedded into the surface of the microspheres. This phenomenon may be explained by the Pickering emulsion theory, specifically, iron oxide nanoparticles existed at the O/W interface to assist the surfactant to form a stable O/W emulsion^11^,^29^. The amount of loaded SPIONs^LA^ seems to have no obvious influence on the surface morphology of the obtained microspheres. Furthermore, in order to reveal the internal structure, a cross-section near the center of MMSH was obtained using focused ion beam (FIB) milling. As shown in Fig. 5(e,f), SPIONs^LA^ are observed on the cross-section as well as on the wall of the inner pores of the microspheres. The pores inside the microspheres are due to the fast skin formation during the solvent extraction, which restricts the further shrinkage/densification of the microspheres during solidification^20^. The surface morphology and internal structure proves that SPIONs^LA^ are distributed over the whole matrix of microspheres, which indicates that SPIONs^LA^ are stable in PHBV–DCM solution and are efficiently encapsulated in the PHBV microspheres.

### Particle size and zeta potential

**Figure 6:**
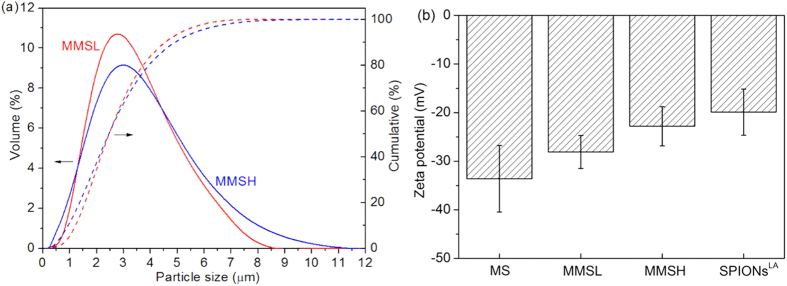
(**a**) particle size distribution of MMSL and MMSH, (**b**) zeta potential of SPIONs^LA^, MS, MMSL and MMSH in water.

The median particle size of both MMSL and MMSH was 2.46 μm (Fig. 6(a)). The *span* (width of particle size distribution) of MMSL and MMSH was 1.37 and 1.62, respectively. Different amounts of encapsulated SPIONs^LA^ seem to have no obvious influence on the mean particle size of prepared microspheres, however higher amount of SPIONs^LA^ slightly increased the microsphere size distribution width. The wider particle size distribution of MMSH can be attributed to the increased instability of the emulsion caused by loading of more SPIONs^LA^. Fig. 6(b) presents the zeta potential of SPIONs^LA^ (−19.9 mV) and microspheres in water. The zeta potential of MS was −33.6 mV, and it increased to −28.1 mV for MMSL and further increased to −22.8 mV for MMSH when more SPIONs^LA^ were encapsulated. This phenomenon is due to the fact that the incorporated SPIONs^LA^ possess a higher zeta potential than MS. The relatively high negative zeta potential of these microspheres is beneficial for their dispersion and stability in aqueous medium.

### Magnetic properties

**Figure 7:**
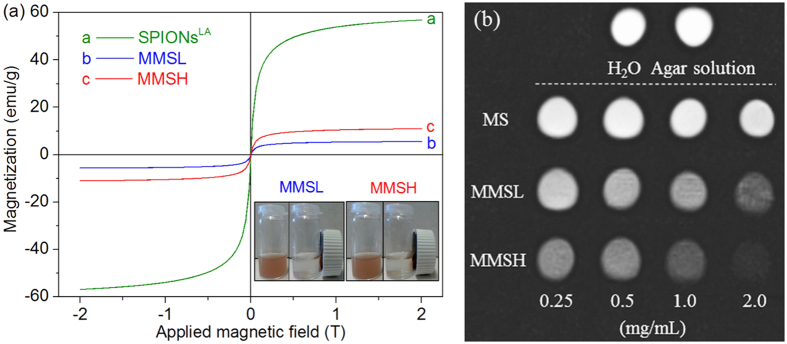
(**a**) magnetization curves measured at room temperature for SPIONs^LA^, MMSL and MMSH. The inset shows the response of MMSL and MMSH to a magnetic field. (**b**) *T*_2_-weighted MR images of H_2_O, 0.1 wt% agar solution and 0.25–2.0 mg/mL of MS, MMSL and MMSH dispersed in 0.1 wt% agar solution.

Fig. 7(a) shows the magnetization curves measured at room temperature for SPIONs^LA^, MMSL and MMSH. The saturation magnetization data obtained at high external field strength (2 T) are an approximation of the theoretical value of saturation magnetization. The magnetization curves, specially for MMSL and MMSH are only slightly increasing at 2 T, which means that the approximation is quite close to the theoretical value. The saturation magnetization values of MMSL and MMSH were 5.6 emu/g and 11.0 emu/g, respectively, and it was 56.8 emu/g for SPIONs^LA^. This behavior is more consistent with the saturation magnetization of bulk maghemite (around 60 emu/g) than with the saturation magnetization of bulk magnetite (around 90 emu/g)^38^. However, it has been shown earlier that dispersions of SPIONs are prone to oxidation during storage, which reduces the saturation magnetization significantly over time, e.g. by up to 28.9%^39^. Although the decrease in saturation magnetization could also derive from the smaller magnetic domain size of SPIONs compared to bulk material, it has been shown earlier that this is not the case for SPIONs^LA^^24^. Therefore it is likely that the particles had oxidized to maghemite already. The inset in Fig. 7(a) demonstrates the dispersion and separation process of the magnetic microspheres. Both MMSL and MMSH were well attractable with a magnet. Therefore, the prepared magnetic PHBV microspheres possessed magnet-induced sensitivity, and this ‘proof of concept’ experiment indicates that they could be utilized as magnetic targeting carriers in biomedical applications.

The prepared magnetic microspheres were qualitatively evaluated for their magnetic contrast capability under MRI scanning. *T*_2_-weighted MR images (Fig. 7(b)) show that MMSL and MMSH produced significant negative contrast effects (signal reductions) in comparison to the control groups, i.e., H_2_O, agar solution and MS in 0.25–2.0 mg/mL. With increasing concentration of microspheres in agar solution, there was a corresponding increase in contrast for MMSL and MMSH. Furthermore, the contrast for MMSH was higher than MMSL at a same concentration due to the higher loading efficiency of SPIONs^LA^ in MMSH. These results show that, although SPIONs^LA^ are encapsulated in PHBV microspheres, they can still be used for magnetic imaging applications.

### Drug release and antibacterial property

**Figure 8:**
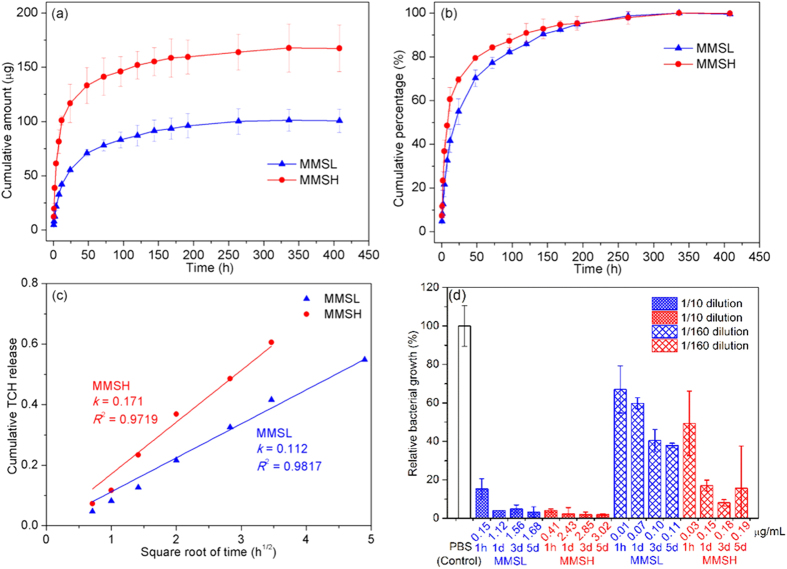
(**a**) Cumulative amount and (**b**) cumulative percentage of TCH release from MMSL and MMSH, (**c**) linear fitting of the cumulative percentage of TCH release versus the square root of time (Higuchi equation), and (**d**) antibacterial activity against *Escherichia coli* (*E. coli*) of diluted TCH solution collected at 1 h, 1 d, 3 d and 5 d.

One of the purposes of this study was to determine if the magnetic PHBV microspheres could serve as potential vehicles to successfully deliver drugs. Fig. 8(a,b) shows the drug release profiles of SPIONs^LA-TCH^ (TCH loaded SPIONs^LA^) loaded PHBV microspheres in PBS. Both MMSL and MMSH exhibited a sustained release of TCH for up to 14 days. Cumulative amounts of 101 μg and 168 μg TCH were released from MMSL and MMSH, respectively. The higher amount of TCH loading in MMSH could explain why it showed a slightly higher initial burst release followed by a relatively faster release at the initial stage compared to MMSL. The mass ratio of TCH in MMSH over that in MMSL is 1.66, which was quite close to the mass ratio (1.62) of the TCH carrier, i.e., SPIONs^LA^ in MMSH over that in MMSL as determined by TGA. The adsorptive affinities of metal oxide nanoparticles for TCH are likely due to several mechanisms, including electrostatic attraction, surface complexation and cation exchange^40^, while the exact mechanism in the present study is yet to be investigated. Phosphate ions exhibit high binding efficiency to the surface of iron oxides^41^, and can thus replace other substances such as drugs which are located on the surface of the SPIONs by the aforementioned mechanism. The fact that some, but not all of the drug is released via burst release could indicate a barrier effect of the polymer towards the drug loaded on iron oxide particles.

PHBV is hydrophobic^42^, and generally degrades quite slowly *in vitro*^27^. Therefore, the drug release from PHBV microspheres is likely to be more dependent on diffusion rather than on polymer degradation^26^,^43^,^44^. The drug release data was fitted to Higuchi equation^45^, and the results are shown in Fig. 8(c). The fitting for both MMSL and MMSH shows good linearity. Thus, TCH release from the magnetic PHBV microspheres can be well described by Higuchi equation, indicating a diffusion controlled release mechanism. In addition, MMSH had a higher kinetic constant (*k*) than MMSL, which confirms that the TCH release from MMSH was faster than from MMSL in the first 24 hours (as shown in Fig. 8(b)).

In order to better differentiate the antibacterial property of TCH solution collected at 1 h, 1 d, 3 d and 5 d during drug release, the solutions were diluted in different ratios. Repetition of the antibacterial test is needed for performing statistical analysis in further study, while the bacterial growth for both MMSL and MMSH (Fig. 8(d)) exhibits an overall decreasing trend when the drug release time increases from 1 h to 5 d, indicating increasing TCH concentration. Moreover, at a given time point, MMSH in general showed less bacterial growth than MMSL, which confirms MMSH released more TCH than MMSL, as shown in Fig. 8(a). Hence, the results of the preliminary antibacterial tests are in good agreement with the results of drug release study and confirm the drug delivery capability of the prepared magnetic PHBV microspheres.

### *In vitro* cytotoxicity

**Figure 9:**
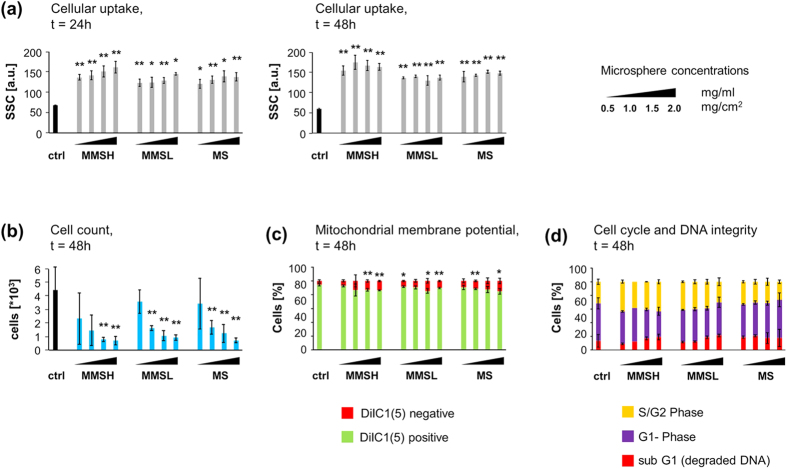
Biocompatibility of MS, MMSL and MMSH on Jurkat T cells. Experiments were performed in triplicates. Results are given as mean ± standard deviations (The asterisks indicate significant differences in Student’s *t*-test compared to the controls *P < 0.05, **P < 0.01). Experiments were performed in 48 well plates (growth area of 1 cm^2^/well) and 1 mL medium. Microsphere concentrations are given in mg/mL and mg/cm^2^. (**a**) SSC increase of viable cells (gated on DiIC_1_(5) positive cells) reflects microsphere uptake by cells; t = 24 h and 48 h, (**b**) relative viable cell count indicates reduced cellular proliferation caused by microspheres; t = 48 h, (**c**) DiIC_1_(5) staining provides information about MMP. DiIC_1_(5) positive cells have an intact MMP and can be considered viable; whereas DiIC_1_(5) negative cells can be considered dying/dead; t = 48 h, (d) PI-triton staining shows that cell cycle and DNA integrity is not influenced by microspheres; t = 48 h.

Jurkat T cells (suspension cells) and HT-29 colon carcinoma cells (adherent cells) were used to investigate the biocompatibility of PHBV microspheres (without TCH) in various concentrations. After 24 h and 48 h of incubation, cells were stained and analyzed in flow cytometry. Staining with the DNA dye Hoechst and differences in size enabled the clear discrimination between cells and microspheres. Using the cyanine dye DiIC_1_(5) (1,1′,3,3,3′,3′-hexamethylindodicarbocyanine iodide), we stained cells with an active mitochondrial membrane potential (MMP) as a measure for intact cell metabolism. Decrease of DiIC_1_(5) stain intensity indicates a disrupted mitochondrial membrane potential. Data were confirmed by AnnexinA5-FITC /propidium iodide staining (not shown), which detects exposure of phosphatidylserine on the outer side of the plasma membrane of apoptotic cells and disrupted plasma membranes of necrotic cells, respectively. Investigating the side scatter (SSC) of viable cells as mean of cellular granularity indicates a concentration dependent cellular uptake of the microspheres after 24 h, whereas after 48h, the SSC values have equaled for all tested concentrations of each microsphere indicating a saturation effect (Fig. 9(a)). The cellular proliferation was dose dependently decreased by the presence of microspheres in Jurkat cells (Fig. 9(b)). In contrast, cell viability, reflected by intact MMP (Fig. 9(c)) and DNA integrity (Fig. 9(d)), were only slightly influenced in Jurkat cells by the microspheres, indicating their biocompatibility even in high concentrations and prolonged incubation times. AnnexinA5-FITC/propidium iodide staining confirmed these results (data not shown). In addition, due to their relatively large particle size, microspheres gradually sedimented, thus at the bottom of the cell culture wells locally even higher microsphere concentrations can be assumed. Therefore, microsphere concentrations are given in both μg/mL and μg/cm^2^.

**Figure 10:**
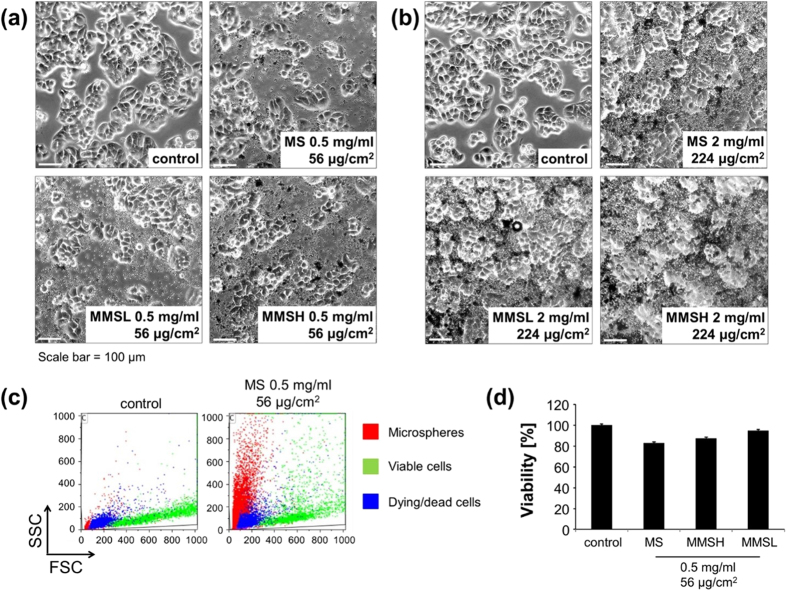
Biocompatibility of MS, MMSL and MMSH on adherent HT-29 cells. Experiments were performed in triplicates, shown are the mean values with standard deviations. HT-29 cells were seeded into cell culture wells (6 well plates; growth area 8.9 cm^2^, 1 mL medium), grown for 24 hours and incubated with microspheres for 24 h. (**a,b**) Transmission microscope images (magnification 10×) of HT-29 cells incubated with (**a**) 0.5 mg/mL microspheres or (**b**) 2 mg/mL microspheres for 24 h. (**c**) Flow cytometry raw data file (Forward scatter versus side scatter) depicts possibility to discriminate between microspheres (red), viable (green) and dying/dead cells (blue) by morphological parameters; (**d**) analysis of cell viability by morphology and mitochondrial membrane potential. Viability of control cells was set to 100%; experiment was performed in triplicates; shown are the mean values with standard deviations.

HT-29 colon carcinoma cells (adherent cells) were incubated with 0.5 mg/mL (56 μg/cm^2^) or 2 mg/mL (224 μg/cm^2^) microspheres (Fig. 10). After 24 h of incubation, transmission microscope images (Fig. 10(a,b)) showed that adherent cells were still tightly attached to the plastic surfaces of the wells and looked phenotypically healthy even in the presence of high microsphere concentrations (Fig. 10(b)). For more detailed investigation, flow cytometry of HT-29 cells was performed after trypsin detachment and staining with DiIC_1_(5) (Fig. 10(c)). As shown by morphological analysis and investigation of mitochondrial membrane potential, incubation with 0.5 mg/mL (56 μg/cm^2^) microspheres induced only a slight loss in cell viability (Fig. 10(d)).

Altogether, cell viability in the presence of MMSL, MMSH as well as MS was better than free SPIONs^LA^ at a comparable concentration as shown in the related previous study^24^, therefore we conclude that encapsulation of SPIONs^LA^ in PHBV microspheres improved their biocompatibility. The biocompatibility of these magnetic PHBV microspheres ensures their potential for use in biomedical applications such as drug delivery and MRI.

## Conclusions

SPIONs after surface modification with lauric acid became much more stable in DCM solution, which therefore allowed the preparation of magnetic SPION-containing PHBV microspheres with high encapsulation efficiency (71.0–87.4%) using emulsion-solvent extraction/evaporation method. The obtained microspheres appeared spherical in shape, and had a median particle size of 2.46 μm. SPIONs^LA^ were observed throughout the matrix of microspheres. Loading efficiencies of the microspheres were determined to be 12.5–20.3 wt%, and the corresponding magnetization was 5.6–11.0 emu/g. The zeta potential of the microspheres increased but it was still negative with increasing content of encapsulated SPIONs^LA^. The developed magnetic PHBV microspheres were readily detectable by MRI, and increasing contrast was observed for the microspheres loaded with higher content of SPIONs^LA^. The magnetic PHBV microspheres were shown to be biocompatible towards both Jurkat cells (suspension cells) and HT-29 cells (adherent cells) even at high concentrations. TCH, as a model drug, was successfully loaded into the microspheres, and released in a diffusion controlled manner. The developed magnetic PHBV microspheres have potential applications in targeted drug delivery and MRI.

## Materials and Methods

### Materials

PHBV with a PHV content of 12 wt% was purchased from Goodfellow (Huntingdon, UK). Polyvinyl alcohol (PVA) (MW ~30,000), Ringer´s solution and Iron (II) chloride tetrahydrate were from Merck (Darmstadt, Germany). DCM, propidium iodide (PI), sodium citrate, triton X-100, acetone and lauric acid were obtained from Sigma-Aldrich (St. Louis, USA). Ammonia solution 25% and Iron (III) chloride hexahydrate were supplied by Roth (Karlsruhe, Germany). TCH was purchased from AppliChem (Darmstadt, Germany). RPMI 1640 medium, fetal calf serum (FCS), Annexin A5-FITC (Ax), Hoechst 33342 (Hoechst) and DiIC_1_ (5) were acquired from Thermo Fisher Scientific (Waltham, USA).

### Preparation of SPIONs and lauric acid-coated SPIONs

Uncoated SPIONs (SPIONs^0^) and lauric acid-coated SPIONs (SPIONs^LA^) were synthesized as described in detail elsewhere^25^. In brief, 1.998 g of iron (II) chloride tetrahydrate and 5.406 g of iron (III) chloride hexahydrate were dissolved in 20 mL of ultrapure water under Ar atmosphere and heated to 80 °C. 16.5 mL ammonia solution (25%) were added to form a blackish precipitate of SPIONs^0^. To obtain SPIONs^LA^, 1.28 g of lauric acid dissolved in acetone were added after that and the suspension was heated to 90 °C for another 15 minutes. Both particle suspensions were purified by dialysis using a molecular weight cut off of 8000 Da and the pH was adjusted to 9.2 using ammonia solution. They were lyophilized and stored at 4 °C until further use.

### Characterization of SPIONs^LA^

#### FTIR measurement

The chemical composition of the lyophilized nanoparticles was determined using FTIR (Nicolet 6700, Thermo Scientific, USA). The sample was mixed with KBr (spectroscopy grade, Merck, Germany) and pressed into pellets. Spectra were recorded in absorbance mode between 4000 and 400 cm^−1^ with a resolution of 4 cm^−1^.

#### Stability of SPIONs^LA^

5 mg SPIONs^0^ and SPIONs^LA^ were placed in a glass vial, respectively. 2 mL DCM were added into the glass vial followed by adding 4 mL water. Photos were taken immediately after adding the water. Afterwards, the mixture was homogenized by a probe sonicator (Branson Sonifier^®^ S-250D, Emerson, USA) at 10% power output for 40 s. Photos were taken 2 min after the sonication. 2 min was chosen here because this is the emulsification time for preparing PHBV microspheres in the following study.

#### TEM

TEM images were taken with a CM 300 UltraTWIN (Philips, Eindhoven, Netherlands) operated at an acceleration voltage of 300 kV. Samples were prepared by drying 10 μL of diluted nanoparticle suspension on a carbon-coated Athene S147-2 copper grid (Plano, Wetzlar, Germany). Particle size distribution of the iron oxide cores was determined using Image J (NIH, USA), and more than 100 particles were analyzed.

### Preparation of magnetic PHBV microspheres

A solid-in-oil-in-water (S/O/W) emulsion-solvent extraction/evaporation method was used to prepare the magnetic PHBV microspheres by modifying the method used in our previous study^43^. Briefly, SPIONs^LA^ (S phase) were added into 3 mL of 3% w/v PHBV–DCM solution (O phase). Two mass ratios of SPIONs^LA^ to PHBV were chosen, i.e., 1:6 (MMSL) and 1:2.5 (MMSH). The mixture was homogenized by a probe sonicator at 10% power output for 40 s. Then, the homogeneous mixture was added into 75 mL of 2% w/v PVA solution (W phase) and emulsified using a homogenizer (T18, IKA, Germany) at 10500 rpm for 2 min. Afterwards, the obtained emulsion was added into 225 mL of 1% w/v PVA solution. The final solution was stirred at 600 rpm for 2 h by an over-head stirrer (Eurostar 20, IKA, Germany) to evaporate the organic solvent. The microspheres were collected by centrifugation (Centrifuge 5430R, Eppendorf, Germany) at 5000 rpm for 4 min, washed in deionized water, and lyophilized (Alpha 1-2 LDplus, Martin Christ, Germany). The microspheres were stored in a desiccator until further use. Pure PHBV microspheres were prepared following the procedure described above, except that no SPIONs^LA^ were added into the PHBV solution.

### Characterization of magnetic PHBV microspheres

#### Surface morphology and internal microstructure

Surface morphology was observed using SEM (LEO 435 VP, Cambridge, UK and Ultra Plus, Zeiss, Germany). Dried microspheres were mounted onto stubs using carbon tapes. No metallic coating was applied on the microspheres for SEM observation. The cross-sections of single microsphere were obtained using a FIB (Helios NanoLab™ 660, FEI, Hillsboro, USA). The microspheres were sputter coated first with 200 nm thick carbon, then with 2 μm thick platinum. A Ga^+^ beam of 30 kV between 40 and 90 pA was used to cut the microspheres. Once the samples had been polished to the desired cross-sections, SEM images were taken in secondary electron imaging mode.

#### Particle size and zeta potential

Particle size and zeta potential of the microspheres were analyzed by Mastersizer particle size analyzer 2000 and Malvern Zetasizer Nano ZS (Malvern, Worcestershire, UK), respectively. For these measurements, microspheres were suspended in deionized water. In addition to the median particle size (*D*_50_), the width of particle size distribution (*span*) was also calculated. The *span* is calculated by (*D*_90_−*D*_10_)/*D*_50_, where *D*_*i*_ means *i*% of the particle distribution lies below the size *D*_*i*_.

#### Encapsulation efficiency and loading efficiency

The encapsulation efficiency and loading efficiency of SPIONs^LA^ in magnetic PHBV microspheres were determined by TGA (Q5000, TA Instruments, USA). The samples were heated from 25 °C to 550 °C under nitrogen flow. The heating rate was 10 °C/min. The loading efficiency and encapsulation efficiency were calculated by equations (1) and (2), respectively:


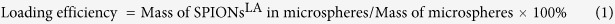



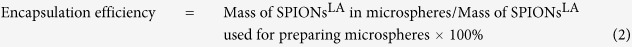


#### Magnetic properties (SQUID and MRI)

The magnetic properties of the samples in solid form (lyophilized powder) were measured using a superconducting quantum interference device (SQUID)-based susceptometer (QD-MPMS-XL-5, Quantum Design Inc, San Diego, USA) in an applied magnetic field of ± 2 T at room temperature. The sample holder used for the measurement was gelatin capsule. Diamagnetic contribution of the sample holder was subtracted. The obtained magnetization is given in emu per gram (of total material). MRI studies were conducted on a 3T Siemens Trio-Tim scanner (Siemens, Erlangen, Germany). Imaging phantoms were prepared by dispersing microspheres in 0.1 wt% agar solution at 0.25–2.0 mg/mL.

#### XRD analysis

The main composition of the microspheres was characterized using XRD (Bruker D8 ADVANCE Diffractometer, Cu *Kα*) at room temperature. Data were collected over the 2*θ* range from 10° to 70° using a step size of 0.014° with 1 second per step.

### *In vitro* drug release study

#### Drug loading

SPIONs^LA^ were soaked in 3 mg/mL TCH–deionized water solution for 24 h. Then, the TCH loaded SPIONs^LA^ (SPIONs^LA-TCH^) were collected by centrifugation, washed in deionized water, and lyophilized. The loading degree of TCH on SPIONs^LA^ was 8.8 wt%. SPIONs^LA-TCH^ loaded PHBV microspheres were prepared following the same procedure as SPIONs^LA^ loaded PHBV microspheres. The drug loading and drug release process were kept off light.

#### Drug release

10 mg MMSL and MMSH were dispersed in 1 mL PBS solution (pH 7.4) and placed in a dialysis bag (Spectra/Por^®^ 1, molecular weight cut-off 6000 to 8000, Carl Roth, Germany), and then immersed in tubes containing 4 mL PBS solution (pH 7.4). The samples were placed in a shaking incubator (90 rpm, 37 °C). After predetermined time points, 1 mL medium was collected and replaced with 1 mL fresh PBS solution. TCH concentration was measured using a UV–Vis spectrophotometer (Specord^®^ 40, Analytik Jena, Germany) at a wavelength of 362 nm. A calibration curve was made with TCH concentration in the range 2.5–100 μg/mL, and the obtained calibration curve follows Beer’s law: *A* = 0.0294 × *C*, where *A* is the absorbance and *C* is the concentration of TCH. The cumulative release (%) of TCH was calculated. The drug release study was carried out for 408 h and performed in triplicate.

#### Drug release kinetics

Higuchi model was used to analyze the TCH release kinetics from the magnetic PHBV microspheres, which is represented by equation: *Q*_*t*_ = *kt*^1/2^, where *Q*_*t*_ is the cumulative percentage of drug release, *k* is the kinetic constant and *t* is the release time^45^. This equation is generally valid for the first 60% of total amount of drug release.

#### Antibacterial test

4 mg MMSL and MMSH were placed in a dialysis bag, respectively, and then immersed in 2 mL PBS solution. 100 μL released medium was collected at 1 h, 1 d, 3 d and 5 d, and diluted in 10 mM sterilized sodium phosphate buffer for antibacterial test. TCH concentration in the released medium was measured using a UV–Vis spectrophotometer. Antibacterial activities of the diluted medium were evaluated using the 96-well plate assay^46^. *E. coli* ATCC 25922 was purchased from Leibniz Institute DSMZ-German Collection of Microorganisms and Cell Cultures (Braunschweig, Germany). *E. coli* were inoculated into fresh nutrient broth (Roth, Karlsruhe, Germany) and incubated at 37 °C for 8 h with shaking to reach the logarithmic phase of growth at a density of 1–5 × 10^7^ cfu/mL. Then, these bacterial suspensions were diluted to a density of 1–2 × 10^5^ cfu/mL. Individual wells of a sterilized 96-well microtitre plate were inoculated with 50 μL of samples, i.e., diluted medium and 50 μL of bacterial suspension. Control wells contained all the components except the samples, which were replaced by 50 μL ampicillin solution of 10 μg/mL (positive control) or 50 μL sterilized sodium phosphate buffer of 10 mM (negative control). The 96-well plates were incubated overnight at 37 °C with shaking. The experiment was conducted in duplicates and the optical density at 600 nm was spectrophotometrically recorded after the incubation by using a temperature-controlled automatic plate reader (Tecan, Männedorf, Switzerland). Percentage of bacterial growth (*P*_bacterial growth_) was determined by


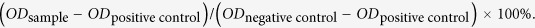


### *In vitro* biocompatibility assays

The non-adherent human T-cell leukemia cell line Jurkat (ACC 282, DSMZ, Braunschweig, Germany) was cultured in RPMI 1640 medium supplemented with 10% FCS. Adherent colon carcinoma HT-29 cells (ATCC/LGC GmbH, Wesel, Germany) were cultured in McCoy’s 5A medium (Gibco^®^, Life Technologies GmbH, Darmstadt, Germany) supplemented with 10% FCS. All cells were cultivated under standard cell culture conditions in a humidified incubator (INCOmed, Schwabach, Memmert, Germany) at 37 °C and 5% CO_2_.

Prior to these experiments, viability and count of cells were determined using a Muse cell analyzer (Merck Millipore, Darmstadt, Germany). Only if the viability exceeded 90% the experiments were continued. The microspheres were sterilized under UV light for 2 h.

Jurkat cells were adjusted to 2 × 10^5^ cells/mL in medium. 1 mL of this suspension, containing the respective concentration of microspheres (without TCH), was seeded into 48 well plates (Greiner Bioone, Frickenhausen, Germany). After 24 h and 48 h, 50 μL aliquots of the cells and microspheres containing suspension were taken and stained for cell viability analysis.

3.4 × 10^5^ HT-29 cells were seeded into each well of a 6 well plate (TPP, Schaffhausen, Switzerland) in medium, grown for 24 h and incubated in the presence of microspheres for further 24 h. Then, transmission optical microscopy was performed using an Axiovert 40 CFL Microscope (Zeiss, Jena, Germany) with a 10x objective with phase contrast. Then, cells were detached with 0.25% trypsin/0.02% EDTA in PBS (PAN Biotech, Aidenbach, Germany) from the wells and resuspended to form single-cell suspensions.

Since microspheres gradually sediment to the bottom of the cell culture wells, indication of microsphere concentration in μg/mL and μg/cm^2^ is required (see Table 1).

Analyses for cell viability were then performed according to a protocol from Munoz *et al*.^47^. For staining, 250 μL of the staining solution (containing 20 μg/mL PI, 0.5 μg/mL Annexin A5-FITC, 10 nM DiI and 1 μg/mL Hoechst 33342 in Ringer´s solution) were added to cell aliquots and incubated for 20 min at 4 °C. The suspensions were then analyzed with a Gallios flow cytometer (Beckman Coulter, Fullerton, USA) for 1 min. Bleed through fluorescence was eliminated by electronic compensation. Forward scatter (FSC) and side scatter (SSC) were considered for morphological analysis of cells. Cell cycle and DNA degradation were examined by propidium iodide-triton (PIT) staining according to the protocol of Nicoletti *et al*.^48^. Briefly, 400 μL of a solution containing 0.1% sodium citrate, 0.1% Triton X-100, and 50 μg/mL PI was added to 50 μL aliquot of cells, incubated overnight at 4 °C in the dark, and nuclear fluorescence was measured in flow cytometry. Data analysis was performed with the software Kaluza 1.2.

### Statistical analysis

Statistical significance analysis was performed using Student’s *t*-test in Microsoft Excel 2010 (Microsoft, Redmond, USA). A value of *P* < 0.05 was considered statistically significant.

## Additional Information

**How to cite this article**: Li, W. *et al*. Facile preparation of multifunctional superparamagnetic PHBV microspheres containing SPIONs for biomedical applications. *Sci. Rep.* **6**, 23140; doi: 10.1038/srep23140 (2016).

## Figures and Tables

**Table 1:** Microsphere concentrations dependent on volume of medium and well size.

Sample	Well area (cm^2^)	Medium (mL)	Microsphere concentration
48 well plate (Jurkat cells)	1	1	1 mg/mL = 1 mg/cm^2^
6 well plate (HT-29 cells)	8.9	1	1 mg/mL = 112 μg/cm^2^
